# Effect of zinc imbalance and salicylic acid co‐supply on *Arabidopsis* response to fungal pathogens with different lifestyles

**DOI:** 10.1111/plb.13344

**Published:** 2021-10-04

**Authors:** M. Quaglia, E. Troni, R. D’Amato, L. Ederli

**Affiliations:** ^1^ Department of Agricultural, Food and Environmental Sciences University of Perugia Perugia Italy

**Keywords:** *Arabidopsis*, *Botrytis cinerea*, *Golovinomyces cichoracearum*, lipid peroxidation, plant defensins, salicylic acid, zinc

## Abstract

- In higher plants, Zn nutritional imbalance can affect growth, physiology and response to stress, with effect variable depending on host–pathogen interaction. Mechanisms through which Zn operates are not yet well known. The hormone salicylic acid (SA) can affect plant ion uptake, transport and defence responses. Thus, in this study the impact of Zn imbalance and SA co‐supply on severity of infection with the necrotrophic fungal pathogen *B. cinerea* or the biotroph *G. cichoracearum* was assessed in *A. thaliana* Col‐0.
- Spectrophotometric assays for pigments and malondialdehyde (MDA) content as a marker of lipid peroxidation, plant defensin 1.2 gene expression by semi‐quantitative PCR, callose visualization by fluorescence microscopy and diseases evaluation by macro‐ and microscopic observations were carried out.
- Zinc plant concentration varied with the supplied dose. In comparison with the control, Zn‐deficit or Zn‐excess led to reduced chlorophyll content and *PDF 1.2* transcripts induction. In Zn‐deficient plants, where MDA increased, also the susceptibility to *B. cinerea* increased, whereas MDA decreased in *G. cichoracearum*. Zinc excess increased susceptibility to both pathogens. Co‐administration of SA positively affected MDA level, callose deposition, *PDF 1.2* transcripts and plant response to the two pathogens.
- The increased susceptibility to *B. cinerea* in both Zn‐deficient and Zn‐excess plants could be related to lack of induction of *PDF 1.2* transcripts; oxidative stress could explain higher susceptibility to the necrotroph and lower susceptibility to the biotroph in Zn‐deficient plants. This research shows that an appropriate evaluation of Zn supply according to the prevalent stress factor is desirable for plants.

## Graphical abstract

Imbalances in zinc supply, with or without salicylic acid co‐supply, alter the *Arabidopsis thaliana* Col‐0 susceptibility to necrotrophic and biotrophic fungal pathogens.

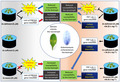

## INTRODUCTION

Several transition metals, including zinc (Zn), are essential micronutrients for living organisms, including higher plants (Mousavi 2011). In plants, Zn is a component of structural proteins and enzymes; in particular, it is the only metal associated with all enzyme classes, mainly anhydrases, dehydrogenases, oxidases and peroxidases, and plays a crucial role in their synthesis and activity (Coleman 1998; Kraemer & Clemens 2005; Mousavi 2011; Cabot *et al*. 2019). According to the Arabidopsis Information Resource (TAIR) database, in *A*. *thaliana* Zn is a component of over 1200 proteins (Kraemer & Clemens 2005). Cell multiplication, auxin synthesis and imbalance, synthesis of nucleic acids, ribosome formation, uptake of micro‐ and macro‐nutrients, water absorption and responses to several stress factors are among the functions in which Zn is involved in plants (Kraemer & Clemens 2005; Duffy 2007; Cabot *et al*. 2019). A positive correlation has been reported between Zn level in plants and in the soil (Wong *et al*. 2019), as well as both positive and negative interactions between Zn and other macro‐ and micro‐nutrients (Mousavi 2011).

Given its importance, imbalances in Zn nutrition, both deficit and excess, can affect plant growth and physiology and alter plant response to biotic and abiotic stresses (Alloway 2008). Zinc deficiency is among the main plant nutrition deficiencies worldwide (Duffy 2007). Symptoms of Zn deficiency appear first on the youngest leaves as discoloration between veins in the form of bleaching, purpling or yellowing, depending on plant species; also, leaf size and internode length are negatively affected (Duffy 2007; Mousavi 2011). Leaf growth is also compromised by excess Zn, which at high concentrations has toxic effects connected to an increase in free radicals and a decrease in activity of the photosynthetic apparatus and ATP synthesis (Mousavi 2011).

Considering biotic stresses, the effect of Zn level on plant response can vary according to the host–pathogen interaction (Duffy 2007; Cabot *et al*. 2019). Thus, with respect to biotrophic fungal pathogens, inadequate Zn nutrition increased susceptibility of pecan trees to *Mycosphaerella dendroides* (Schwein.) Demaree & Cole and *Cercospora fusca* F.V. Rand (Moznette et al. 1940), as well as susceptibility of rubber trees to *Oidium* spp. (Bolle‐Jones & Hilton 1956). In contrast, Zn‐deficient soybean plants showed less susceptibility to *Phakopsora pachyrhizi* Syd. & P. Syd. (Helfenstein *et al*. 2015). An increase in susceptibility to the biotroph *Erysiphe graminis* DC. was observed in Zn‐supplied wheat cultivars (Bolle‐Jones & Hilton 1956).

Under Zn stress, application of the phytohormone salicylic acid (SA) may improve plant growth and alleviate damage due to Zn toxicity (Es‐sbihi *et al*. 2020). Exogenous SA has also been reported to play an important role in ion uptake and transport (Shi & Zhu 2008; Sharma *et al*. 2020) and in nutrient fortification (Smoleń *et al*. 2016). Moreover, SA has positive effects on the response to both biotic and abiotic stresses, including metal toxicity (Spletzer & Enyedi 1999; Mandal *et al*. 2009; Gondor *et al*. 2016).

In the host–fungus interaction, Zn level affects not only the plant but also the attacker. Indeed, several studies have shown that Zn is involved in phytopathogenic fungal growth, sexual morphogenesis, sporulation, spore germination and synthesis of toxins and mycotoxins (Duffy 2007).

Zinc level in plants is also important for human health, vegetables being among Zn sources in the human diet. However, some vegetables, such as cereals and legumes, although rich in Zn, are also rich in phytate, which inhibits Zn absorption in the intestinal lumen (Brown *et al*. 2001).

In this research, we investigated the effect of variations in Zn dose, supplied as zinc sulphate, at physiological dose, excess or absent (deficiency), on *Arabidopsis thaliana* Col‐0 physiological and morphological parameters (pigment level, lipid peroxidation, callose deposition) and on the severity of infections with two pathogens with different lifestyles: the necrotroph *Botrytis cinerea* Pers. ex Fr. and the biotroph *Golovinomyces* *cichoracearum* (DC.) V.P. Heluta. In *A. thaliana*, the infection process of both pathogens begins with conidia germination, through phases of penetration and colonization and end with sporulation, consisting in the formation of a new inoculum. Both pathogens penetrate the intact cuticle of the host by formation of an appressorium and penetration peg (Williamson *et al*. 2007; Micali *et al*. 2008). The biotrophic colonization of *G. cichoracearum* proceeds with the formation of haustoria, specialized organs through which the fungus assimilates nutrients from living cells of the host, with which it establishes a trophic relationship (Szabo & Bushnell 2001). Production of cell wall apposition in the form of papillae and encasement of haustoria in papillae extensions, both containing callose, have been observed in *A. thaliana* during *G. cichoracearum* penetration and colonization, respectively, as a plant defence response (Micali *et al*. 2008). Increased resistance to the pathogen by induction of callose deposition requires exogenous treatment, including SA treatment (Wang *et al*. 2013). The necrotroph *B. cinerea* kills cells during colonization through a plethora of enzymes, toxins and other compounds, such as oxalic acid, and feeds on dead plant tissues (Williamson *et al*. 2007; Jiang *et al*. 2016). Moreover, *B*. *cinerea* can trigger programmed cell death (PCD) of host cells (Williamson *et al*. 2007). Under controlled laboratory conditions, both pathogens complete the infection cycle on *A. thaliana* in approximately in 4–5 days (Williamson *et al*. 2007; Micali *et al*. 2008; Jiang *et al*. 2016).

Since, the phytohormone SA is involved in *A. thaliana* resistance to both *B. cinerea* (Ferrari *et al*. 2003) and *G. cichoracearum* (Reuber *et al*. 1998) and, as reported above, to influence mineral uptake and plant response to different metal levels, we here assessed the effect of Zn and SA co‐supply on the above parameters.

## MATERIAL AND METHODS

### Plants and treatments

Seeds of *A*. *thaliana* L. Heynh. wild‐type Columbia (Col‐0) were surface‐sterilized in 70% (v/v) ethanol and 7% (v/v) sodium hypochlorite, rinsed in sterile distilled water then vernalized in sterile distilled water at 4 °C in the dark for 48 h. With minor modifications to Zeng *et al*. (2018), a sterilized woody toothpick was used for each seed sown in 0.5 ml sterilized tubes containing 150 µl 0.7% w/v water agar (Agar Bios Special LL; Biolife Italiana, Milan, Italy) and filled with 1/2 Hoagland nutrient solution. Tubes were kept in a growth chamber at 22 °C/20 °C, day/night, 60–75% relative humidity and 14‐h light photoperiod at a photosynthetic photon fluence rate of 120 μmol m^−2^ s^−1^ supplied by daylight lamps (Powerstar HQI‐T 400 W/D daylight lamp; Osram) and fluorescent lamps (Philips TLD, the Netherlands). Two weeks after sowing, plants were moved to buckets filled with nutrient solution that was replaced every 2 weeks.

Treatments were provided to each bucket as water solution of zinc sulphate (ZnSO_4_; Merck, Darmstadt, Germany) at 2 µM (Zn‐sufficient), 25 µM (Zn‐excess) or water without ZnSO_4_ (Zn‐deficit). The concentration 25 µM was chosen as it is reported in the literature to mimic conditions of Zn excess in non‐accumulator *A. thaliana* (van de Mortel *et al*. 2006). These solutions were also applied with added salicylic acid (SA; Merck) at 0.1 mg l^−1^, dose in the range commonly used for exogenous applications to plants. Hoagland solution and treatments were at pH of 6.0. Since we aimed to investigate the response to fungal pathogens, only 1‐week treatments were used to induce Zn deficiency or excess, thus avoiding excess stress and toxicity problems.

### Zinc determination in *Arabidopsis* leaves

Total Zn content was determined according to the US‐EPA Method 3052B (USEPA 1996) in Zn‐deficient, Zn‐sufficient or Zn‐excess plants, co‐treated or not with 0.1 mg l^−1^ SA, at 7 dpt (days post‐treatment). Briefly, plant samples were oven‐dried at 60 °C for 48 h and finely ground in a mixer mill (Retsch MM200; Retsch, Verder Scientific, Bergamo, Italy). For each sample, 0.3 g powder was microwave‐digested in an ETHOS One high‐performance microwave digestion system (Milestone, Sorisole, Bergamo, Italy) with 8 ml ultrapure concentrated nitric acid (65% w/w) and 2 ml hydrogen peroxide (30% w/w). The heating programme for digestion was 30 min at 1000 W and 200 °C. After cooling, the digests were diluted with Milli‐Water (18.2 MΩ) up to 20 ml then filtered using a 0.22‐μm filter. Zinc standard solutions was prepared by diluting the corresponding stock solutions (standards 1000 mg l^−1^ for AAS TraceCert) with HPLC‐grade water. Zinc was then determined using a Shimadzu AA‐6200 atomic absorption spectrophotometer (Shimadzu, Tokyo, Japan). The accuracy was validated using a recovery test (n = 3) by adding a Zn standard solution (4 mg l^−1^) into a mixture of a Zn‐enriched sample and nitric acid prior to digestion in tubes and after appropriate dilution according to the above reported US‐EPA method. The experiment was independently repeated three times with three plants per treatment.

### Pigment determination

Leaf tissue (100 mg FW) taken at 7 dpt from Zn‐deficient, Zn‐sufficient or Zn‐excess plants, with or without 0.1 mg l^−1^ SA, was ground in a mortar with a pestle and pigments extracted with 1.5 ml 80% acetone. The obtained samples were centrifuged at 10 000 g for 6 min and the supernatant collected for spectrophotometric assay. Pigment absorption was recorded at 663, 645 and 470 nm with a UV‐Visible spectrophotometer (V‐1200, Chrom Tech, Apple Valley, MN, USA). Total chlorophylls were calculated using the formula of Arnon (1949) and carotenoids as reported by Lichtenthaler & Wellburn (1985). Each experiment was independently repeated three times, with three plants per treatment and two leaves per plant.

### Lipid peroxidation

Lipid peroxidation was measured in *A. thaliana* leaves taken at 7 dpt from Zn‐deficient, Zn‐sufficient or Zn‐excess plants, with or without 0.1 mg l^‐1^ SA, using the malondialdehyde (MDA) assay (Heath & Packer 1968). Leaf samples (200 mg FW) were homogenized with 1.5 ml 0.1% trichloroacetic acid (TCA), centrifuged at 10 000 g for 20 min and 1.0 ml 0.5% thiobarbituric acid (TBA) in 20% TCA added to a 0.5 ml aliquot of the supernatant. After incubation at 95 °C for 30 min, the mixture was immediately cooled on ice, centrifuged at 10 000 g for 10 min then absorbance recorded at 532 and 600 nm with the above UV‐Visible spectrophotometer. MDA content was calculated using an extinction coefficient of 155 mM^−1^ cm^−1^ by subtracting non‐specific absorption at 600 nm from absorption at 532 nm. Each experiment was independently repeated three times, with three plants per treatment and two leaves per plant.

### Pathogens, inoculations and disease evaluations

Two fungal pathogens with different lifestyles were chosen: *Botrytis cinerea*, necrotrophic agent of grey mould, and *Golovinomyces cichoracearum* (formerly *Erysiphe cichoracearum* D.C.), biotrophic agent of powdery mildew. Leaf inoculations were performed using the isolates and methods reported in Ederli *et al*. (2015) at 7 dpt.

Briefly, a pre‐germinated conidial suspension of *B. cinerea* at 1 × 10^5^ conidia ml^−1^ in 10 mM sucrose and 10 mM KH_2_PO_4_ added to 0.04% (v/v) Tween^®^ 20 [10% (v/v) aqueous solution; Boehringer Mannheim, Germany] was drop‐inoculated placing two drops of 5 µl each per leaf. For *G. cichoracearum*, a conidial suspension of 1 × 10^6^ conidia ml^−1^ in sterile deionized water and 0.04% (v/v) Tween^®^ 20 was spray‐inoculated until runoff using a hand atomizer. Inoculated plants were incubated in the conditions described for plant growth but with 100% RH, by covering the plants with cellophane.

Disease evaluation was carried out on inoculated leaves 5 days post‐inoculation (dpi) for *B. cinerea* and 7 dpi for *G. cichoracearum*. Following Ederli *et al*. (2015), for *B. cinerea* necrotic lesion diameter was measured, while for *G. cichoracearum* total number of colonies, conidiophores per colony and conidia per colony were counted under a light microscope (Carl Zeiss, Jena, Germany) at 5X magnification after clearing in 96% (v/v) ethanol at 60 °C × 15 min and staining with Trypan blue solution [0.01% Trypan blue (v/v); Merck, Darmstadt, Germany) in lactic acid:phenol:glycerol:water (1:1:1:1 v/v/v/v) (Reuber *et al*. 1998).

Each experiment was independently repeated three times with: for *B. cinerea*, five plants per treatment and five leaves per plant with each leaf drop inoculated at two places, for a total of 50 lesions per treatment; for *G. cichoracearum* inoculation, six leaves per treatment and six randomly selected areas (2.5 mm^2^) per leaf, for a total of 0.15 cm^2^ per leaf.

### Extraction of RNA and PCR analysis

Leaf samples were collected from plants that were Zn‐deficient, Zn‐sufficient or Zn‐excess, with or without 0.1 mg l^−1^ SA, at 7 dpt. Samples were snap‐frozen in liquid nitrogen and stored at −80 °C. Total RNA was extracted and DNase treatment performed using the PureLink^TM^ RNA Mini Kit and PureLink^TM^ DNase Set (Ambion, Life Technologies, Carlsbad, CA, USA), respectively, according to the manufacturer’s protocols. For each sample, total RNA was measured with an Qbit^TM^ 3.0 Fluorometer (Thermo Fisher Scientific, Rodano, Milan, Italy) and 500 ng total RNA was reverse‐transcribed using PrimeScript^TM^ RT‐PCR Kit (Takara, Saint‐Germain‐en‐Laye, France) according to the manufacturer’s protocol.

Semiquantitative PCR was performed using the T100^TM^ Thermal Cycler (BioRad, Foster City, CA, USA) in a total volume of 20 µl containing 30 ng DNA template, TB Green^®^ Premix Ex Taq^TM^ II (Takara) and 10 µM forward and reverse primers designed for the plant defensin 1.2 (*PDF 1*.2) *At5g44420*, or for the elongation factor 1‐α (*EF 1‐α*) *At1g07940*, the latter used as housekeeping gene. Primer sequences (Ederli *et al*. 2020) were as follows: *PDF 1.2* forward 5’‐TTTGCTGCTTTCGACGCAC‐3’ and reverse 5’‐TAACATGGGACGTAACAGATA‐3’; *EF‐1α* forward 5’‐AAGGAGGCTGCTGAGATGAA‐3’ and reverse 5’‐ TGGTGGTCTCGAACTTCCAG‐3’.

The PCR programme for *PDF 1.2* was as reported in Ederli *et al*. (2021) with slight modifications. Briefly, it consisted of 94 °C for 1 min 15 s, 32 cycles of 5 s at 94 °C, 20 s at 57.5 °C and 45 s at 72 °C, followed by 6 min at 72 °C. For *EF 1‐α*, the PCR programme differed for cycles number (40) and annealing temperature (60 °C). Amplification products (190 bp for *PDF 1.2* and 120 bp for *EF1‐α*) were separated on 1.2% agarose gels in 0.5x Tris‐acetate‐EDTA (TAE) buffer at 100 V for 20 min, stained with ethidium bromide and visualized with a UV transilluminator. The authenticities of the PCR products were checked by two‐directional sequencing using an ABI Prism 310 genetic analyser (Perkin Elmer Life and Analytical Sciences). Band intensities were determined on a scanned filter using ImageJ software (Schneider *et al*. 2012). Levels of transcripts are expressed as relative amounts. The experiment was independently repeated three times with three biological replicates from two leaves per plant of three individual plants per treatment.

### Callose quantification

According to Quaglia *et al*. (2017), callose deposition was quantified using an epifluorescence microscope equipped with UV filters (excitation, BP 365‐395; barrier, LP 420) and ImageJ software (Schneider *et al*. 2012) on leaves at 7 dpt from Zn‐deficient, Zn‐sufficient or Zn‐excess plants, with or without 0.1 mg l^−1^ SA, after clearing by immersion for 10 min in 96% ethanol at 80 °C and staining with aniline blue (Carlo Erba Reagents, Italy). The experiment was independently repeated three times, with three plants per treatment, two leaves per plant and two photographs (area 2.5 mm^2^) taken at random for each leaf.

### Statistical analysis

Data on the effects of the different Zn doses and SA co‐supply on leaf total chlorophyll, carotenoids, MDA, *PDF1.2* gene transcripts, callose, Zn and infection severity were separately submitted to two‐way (zinc dose × SA treatment) ANOVA and significant differences compared by Duncan multiple range test (*P* = 0.05), using the Excel extension DSAASTAT (Onofri & Pannacci 2014). Details on experimental protocols are given in the figure legends.

## Results

### Effect of differences in Zn and SA supply on leaf Zn content

**Fig. 1:**
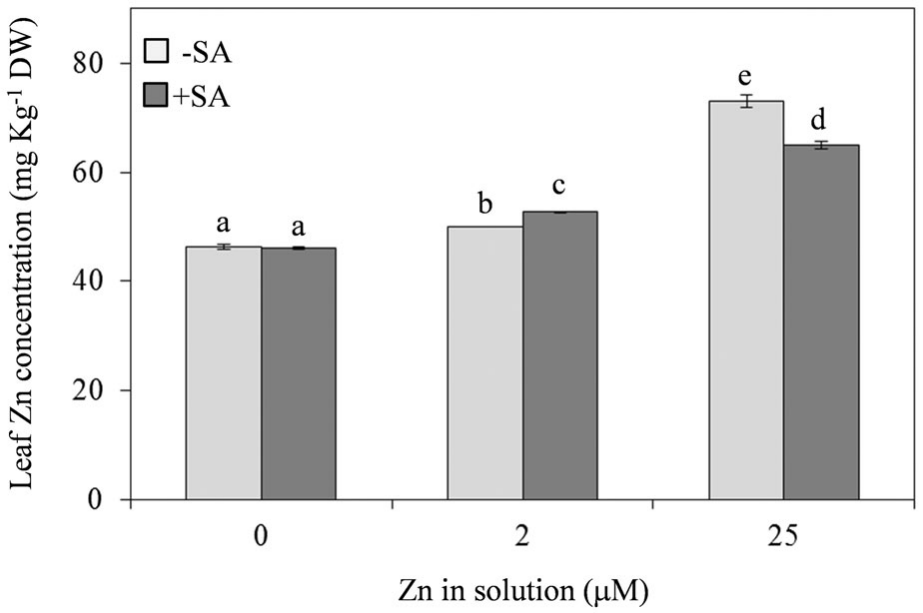
Zinc (Zn) concentrations in leaves of *A*. *thaliana* Col‐0 supplied with water solution of zinc sulphate at 2 µM (zinc‐sufficiency) or 25 µM (zinc‐excess) or water solution without zinc sulphate (zinc‐deficiency), and the same solutions with 0.1 mg l^−1^ salicylic acid (SA) added 7 days post‐treatment. The experiment was independently repeated three times, with three plants per treatment. Data were submitted to two‐factor (zinc dose × SA treatment) ANOVA. Each column represents mean (n = 3) ± SE. Different letters indicate significant differences (*P* ≤ 0.05; Duncan multiple range test).

With respect to the physiological Zn dose (2 µM), a decrease or increase in Zn concentration in the nutrient solution resulted in significant reductions or increases of Zn content in *A. thaliana* leaves at 7 dpt, respectively (Fig. 1). In particular, Zn deprivation corresponded to only a slight decrease (−8%) in Zn accumulation in leaves, while Zn excess produced a strong increase (+46%) in Zn leaf concentration (Fig. 1).

Co‐administration with SA did not affect the Zn content in plants supplied with Zn‐deficient solution, while there was a significant, but weak, increase (+5%) in Zn concentration in plants treated with the physiological Zn dose, and a significant reduction in Zn content (−11%) in plants with excess Zn supply (Fig. 1).

### Effect of differences in Zn and SA supply on photosynthetic pigments and lipid peroxidation

**Fig. 2:**
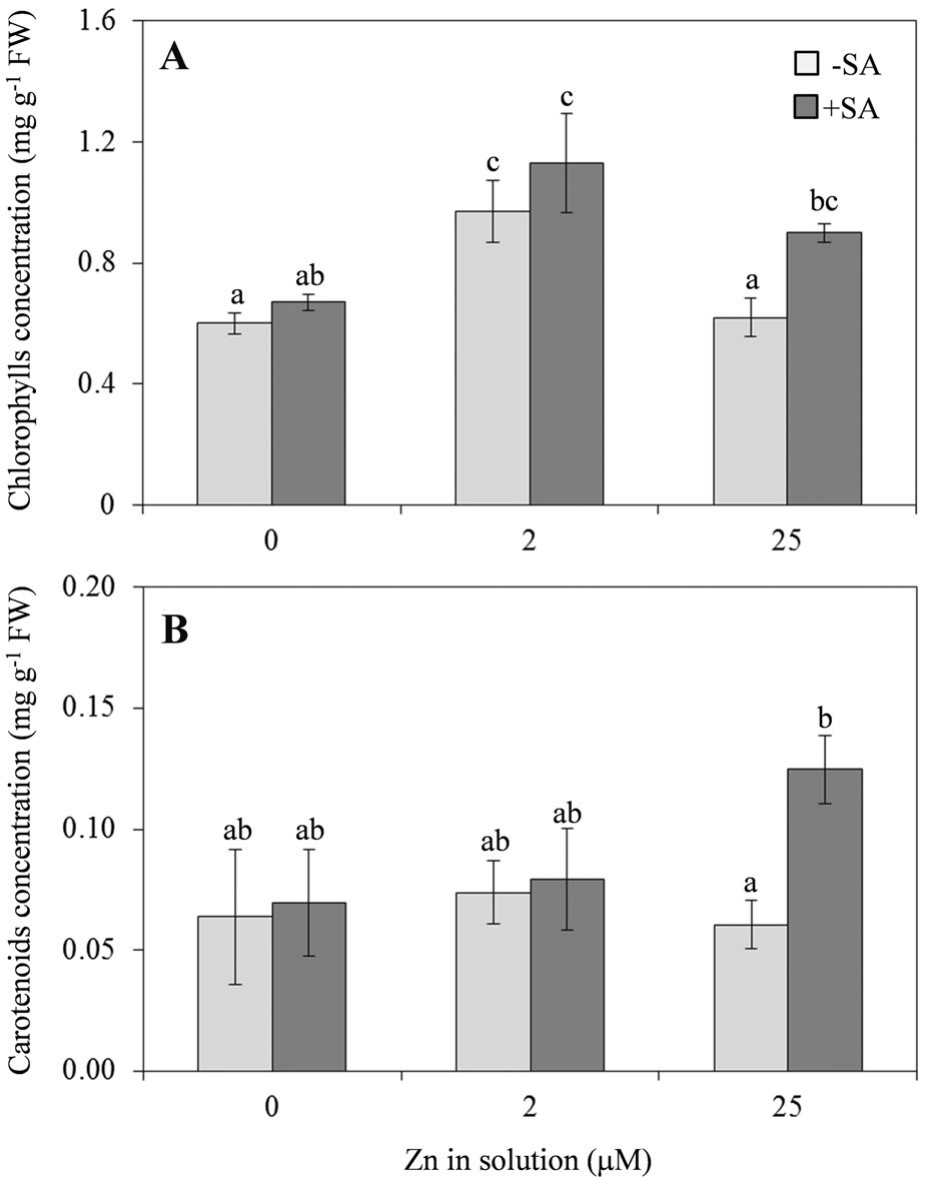
Total chlorophyll (A) and carotenoid (B) content in leaves of *A*. *thaliana* Col‐0 supplied with water solution of zinc sulphate at 2 µM (zinc‐sufficiency) or 25 µM (zinc‐excess) or water solution without zinc sulphate (zinc‐deficiency), and the same solutions with 0.1 mg l^−1^ salicylic acid (SA) added 7 days post‐treatment. Each experiment was independently repeated three times, with three plants per treatment. Data were submitted to two‐factor (zinc dose × SA treatment) ANOVA. Each column represents mean (n = 3) ± SE. Different letters indicate significant differences (*P* ≤ 0.05; Duncan multiple range test).

In plants supplied with Zn‐deficient or Zn‐excess solution, the total chlorophyll content at 7 dpt was significantly lower (−37% approximately) than that in plants supplied with Zn solution at physiological concentration (Fig. 2A). The different Zn doses did not have marked effects on the carotenoid content (Fig. 2B). However, according to van de Mortel *et al*. (2006), at the time of analysis (7 dpt), no visible chlorosis was observed in these plants (data not shown). At the same time, there was a general tendency towards an increase in chlorophyll and carotenoid content in all samples as a consequence of simultaneous SA supply, even if the differences between SA‐supplied or SA‐deprived samples were significant only under excess Zn conditions (Fig. 2A and B).

**Fig. 3:**
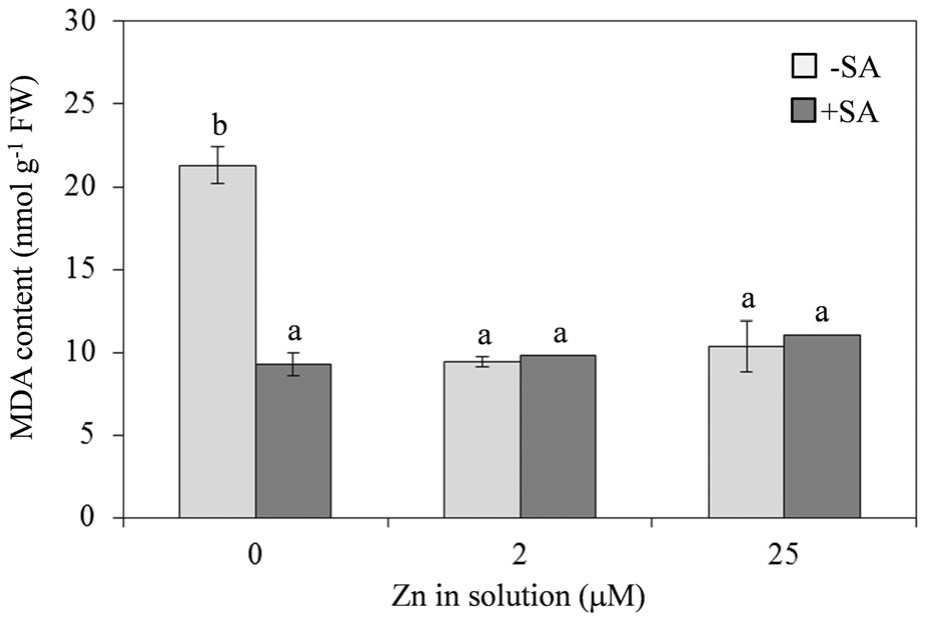
Malondialdehyde (MDA) content in leaves of *Arabidopsis thaliana* Col‐0 supplied with water solution of zinc sulphate at 2 µM (zinc‐sufficiency) or 25 µM (zinc‐excess) or water solution without zinc sulphate (zinc‐deficiency), and the same solutions with 0.1 mg l^−1^ salicylic acid (SA) added 7 days post‐treatment. Each experiment was independently repeated three times, with three plants per treatment. Data were submitted to two‐factor (zinc dose × SA treatment) ANOVA. Each column represents mean (n = 3) ± SE. different letters indicate significant differences (*P* ≤ 0.05; Duncan multiple range test).

As regards oxidative stress, in comparison with plants supplied with Zn solution at physiological concentration, the MDA content, an indicator of lipid peroxidation, increased significantly (+125%) in plants supplied with Zn‐deficient solution, while there were no changes in plants under excess Zn conditions (Fig. 3). Added SA did not affect the MDA content in plants supplied with physiological or excess Zn, while MDA values of plants supplied with Zn‐deficient solution were similar to levels observed in plants supplied with Zn solution at physiological concentration (Fig. 3).

### Effect of differences in Zn and SA supply on *Arabidopsis* susceptibility to *B. cinerea* and expression of *PDF 1.2*

**Fig. 4:**
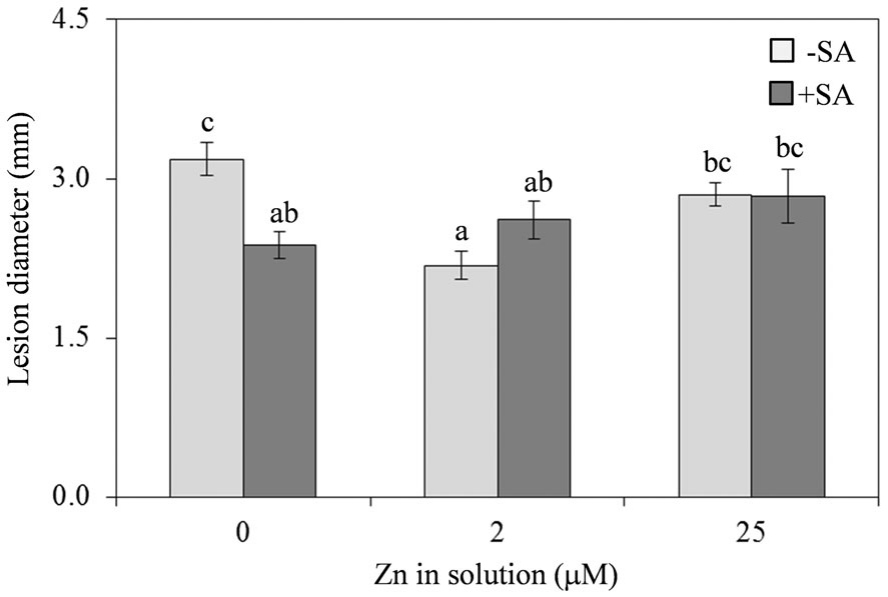
Lesion diameter on leaves of *Arabidopsis thaliana* Col‐0 plants supplied with water solution of zinc sulphate at 2 µM (zinc‐sufficiency) or 25 µM (zinc‐excess) or water solution without zinc sulphate (zinc‐deficiency), and the same solutions with 0.1 mg l^−1^ salicylic acid (SA), 5 days post‐inoculation with conidial suspension of *Botrytis cinerea*. Each experiment was independently repeated three times with five plants per treatment and five leaves per plant and each leaf drop inoculated at two points, with 50 lesions measured per treatment. Data from the three experiments were submitted to two‐factor (zinc dose × SA treatment) ANOVA. Each column represents mean (n = 3) ± SE. Different letters indicate significant differences (*P* ≤ 0.05; Duncan multiple range test).

Zinc supply beyond the physiological range increased *A. thaliana* Col‐0 susceptibility to the necrotrophic fungal pathogen *B. cinerea* (Fig. 4). Instead, plants supplied with Zn‐deficient or Zn‐excess solutions had much larger average diameter of necrotic lesions produced by the pathogen than plants supplied with Zn solution at physiological concentration (Fig. 4). These differences were particularly pronounced among plants supplied with Zn‐deficient solution and Zn solution at physiological level (Fig. 4). Moreover, only in plants supplied with Zn‐deficient solution the co‐administration of SA significantly reduced lesion diameter (Fig. 4).

**Fig. 5:**
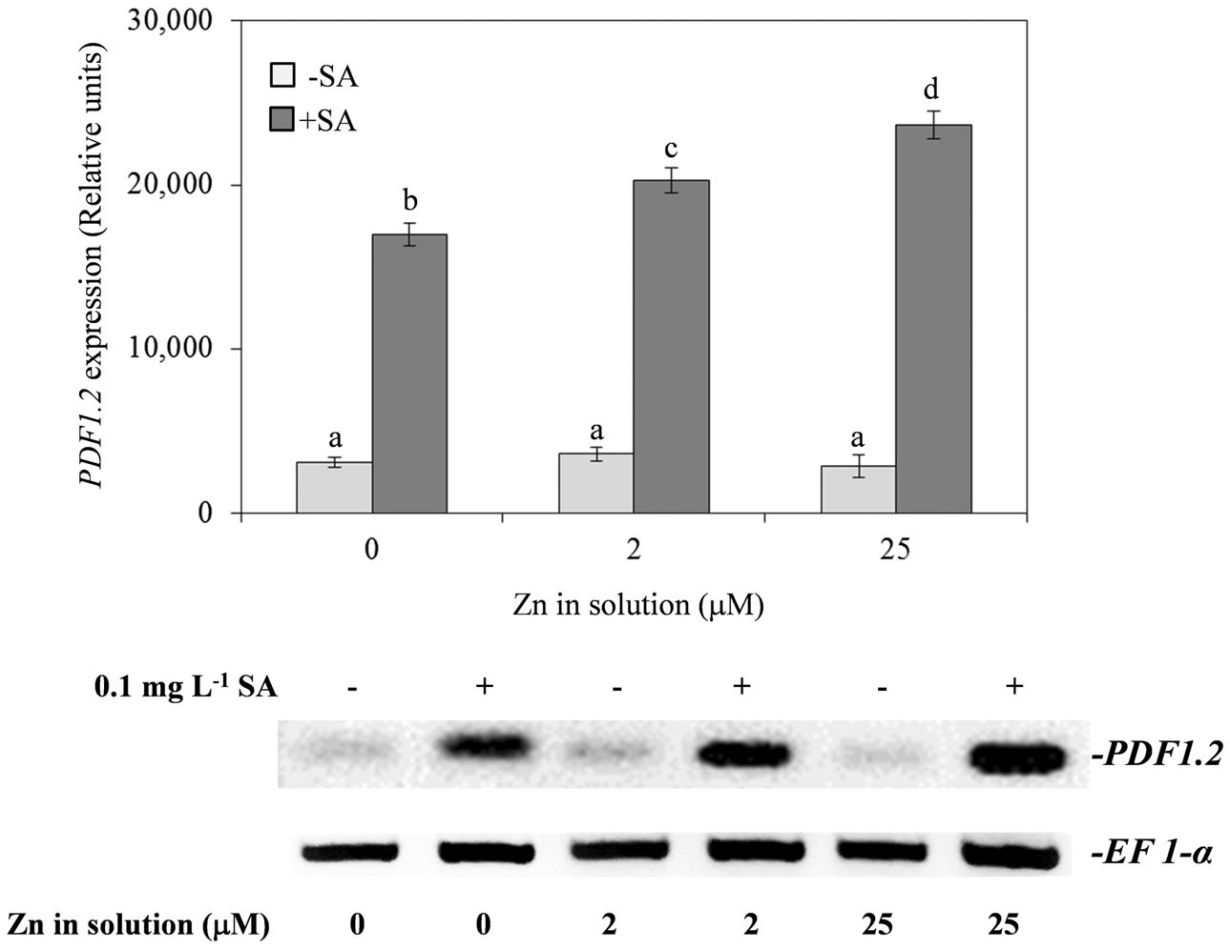
*PDF 1.2* gene transcript accumulation in leaves of *Arabidopsis thaliana* Col‐0 plants supplied with water solution of zinc sulphate at 2 µM (zinc‐sufficiency), 25 µM (zinc‐excess) or water solution without zinc sulphate (zinc‐deficiency), and the same solutions with 0.1 mg l^−1^ salicylic acid (SA), at 5 days post‐inoculation with conidial suspension of *Botrytis cinerea*. Semi‐quantification of mRNA levels loaded in each line was performed by co‐amplification and normalization with an internal standard (Elongation factor‐1α; *EF 1*‐α). Relative intensities of signals (mean ± SE) were measured using image analysis software ImageJ. Each experiment was independently repeated three times, with three plants per treatment and two leaves per plant. Data were submitted to two‐factor (zinc dose × SA treatment) ANOVA. Different letters indicate significant differences (*P* ≤ 0.05; Duncan multiple range test). A representative gel is shown below each histogram.

Since *PDF 1.2* expression is associated with resistance to *B. cinerea* (Cabot *et al*. 2013), the transcript level of this gene was assessed. In comparison with plants supplied with Zn solution at physiological concentration, only a weak and not significant decrease in the level of *PDF 1.2* transcripts was observed both in plants supplied with Zn‐deficient and Zn‐excess solutions (Fig. 5). The addition of SA significantly increased *PDF1.2* transcript accumulation in all Zn concentrations with an increase in gene expression that was even larger the higher the Zn administration to plants (Fig. 5).

### Effect of differences in Zn and SA supply on *Arabidopsis* susceptibility to *G. cichoracearum* and callose deposition

**Fig. 6:**
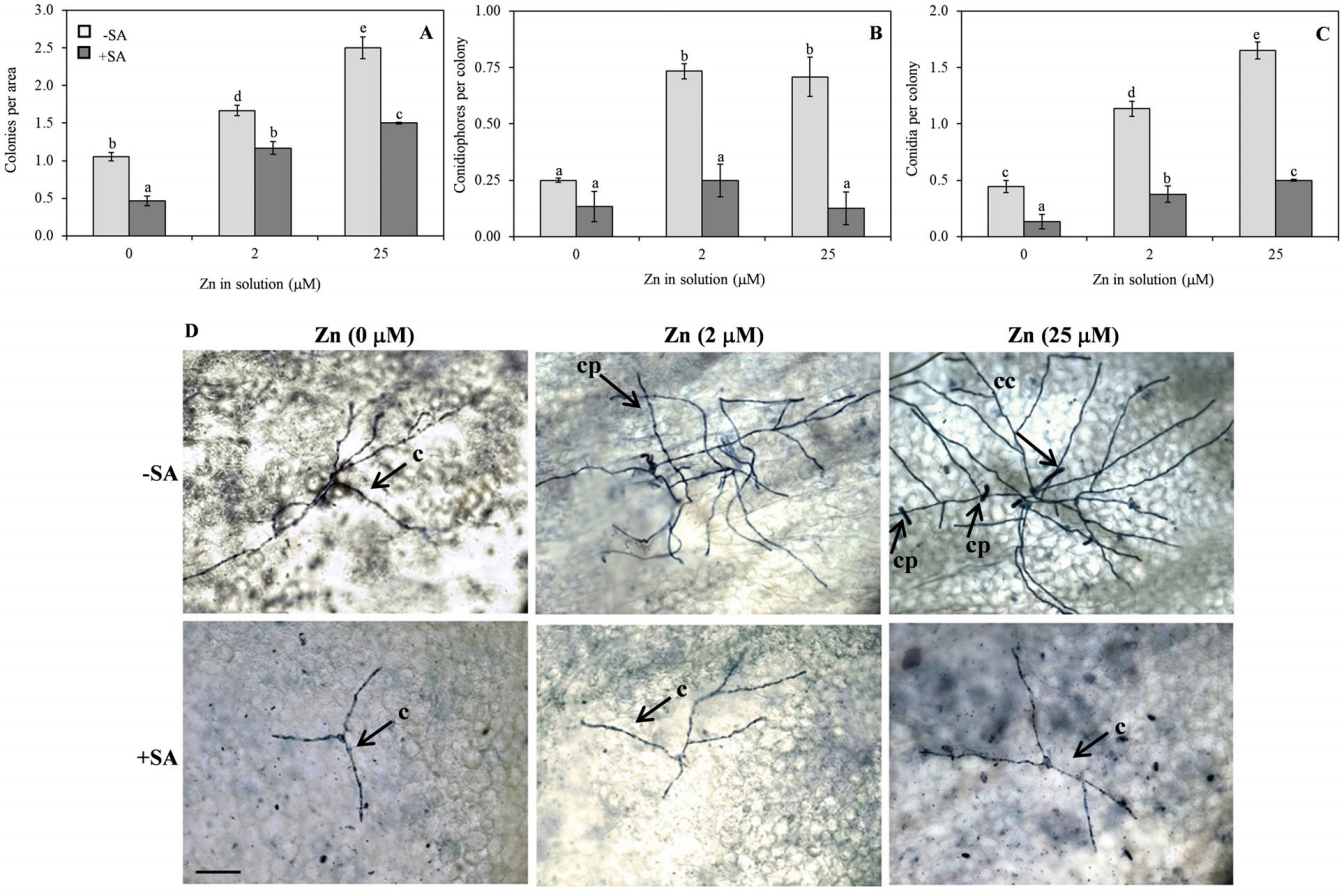
*Golovinomyces cichoracearum* infection in leaves of *Arabidopsis thaliana* Col‐0 plants supplied with water solution of zinc sulphate at 2 µM (zinc‐sufficiency) or 25 µM (zinc‐excess) or water solution without zinc sulphate (zinc‐deficiency), and the same solutions with 0.1 mg l^−1^ salicylic acid (SA), at 7 days post‐inoculation with conidial suspension of the pathogen. Fungal colonies per leaf area (A), conidiophores per colony (B) and conidia per colony (C) were counted. The experiment was independently repeated three times. In each experiment, six leaves per treatment were inoculated and six randomly selected areas (2.5 mm^2^) per leaf was examined, with a total of 0.15 cm^2^ per leaf. Data were submitted to two‐factor (zinc dose × SA treatment) ANOVA. Each column represents mean (n = 3) ± SE. Different letters indicate significant differences (*P* ≤ 0.05; Duncan multiple range test). Representative microscope images of Trypan blue‐stained leaves treated as above (D). Scale bar: 100 µm. Arrows indicate colony without conidiphores (c), conidiophores (cp) and chains of conidia (cc).

**Fig. 7:**
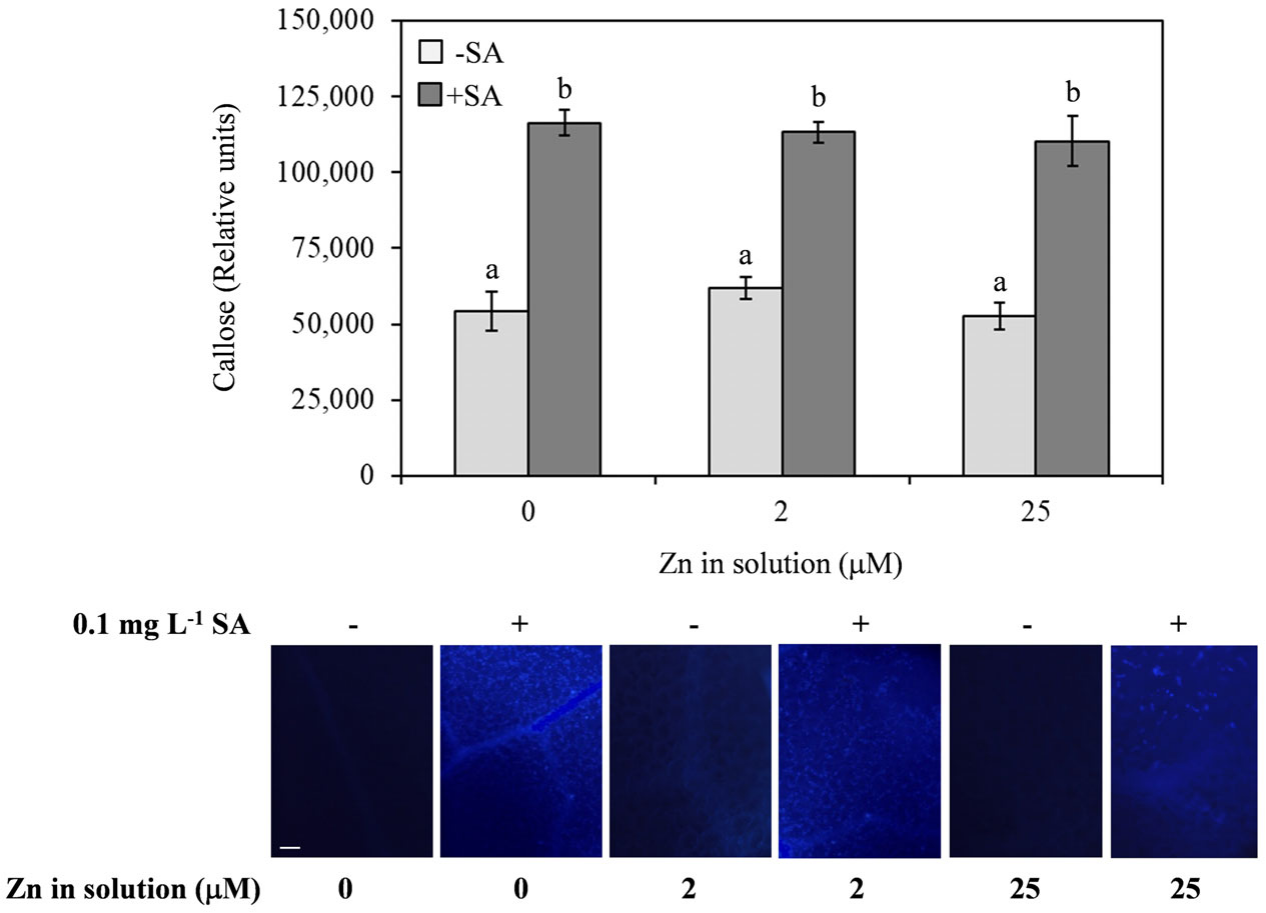
Callose deposition (%) in digitalized photos in leaves of *Arabidopsis thaliana* Col‐0 plants supplied with water solution of zinc sulphate at 2 µM (zinc‐sufficiency) or 25 µM (zinc‐excess) or water solution without zinc sulphate (zinc‐deficiency), and the same solutions with 0.1 mg l^−1^ salicylic acid (SA), 7 days post‐treatment. The experiment was independently repeated three times, with three plants per treatments, two leaves per plant and two photographs taken at random for each leaf. Data from three experiments were submitted to two‐factor (zinc dose × SA treatment) ANOVA. Each column represents mean of 36 replicated photos per treatment ± SE. Different letters indicate significant differences (*P* ≤ 0.05; Duncan multiple range test). Bottom: representative digitalized areas of callose localization in leaves after aniline blue staining (blue fluorescence). Bar: 200 µm.

Susceptibility to *G. cichoracearum* increased with increasing concentrations of Zn. Indeed, as the Zn supply increased from 0 to 25 µM, the number of fungal colonies per leaf area, conidiophores per colony and conidia per colony significantly increased (Fig. 6). Addition of SA negatively affected fungal growth. Indeed, there was a marked reduction in all the considered parameters after SA co‐supply (Fig. 6). Callose deposition was not affected by the different Zn concentrations but was strongly induced after SA co‐supply, irrespective of Zn concentration (Fig. 7).

## DISCUSSION

The impact of mineral nutrition on plant defence against biotic stresses has been investigated in recent years, with Zn as one of the most used micronutrients in these studies (Cabot *et al*. 2019). Zinc nutritional imbalances lead to alterations in plant physiology and changes in response to biotic stresses (Duffy 2007; Helfenstein *et al*. 2015; Cabot *et al*. 2019). The role of Zn in plant protection varies depending on the host–pathogen interaction, and both positive and negative effects on disease severity have been reported (Duffy 2007; Huber *et al*. 2012; Cabot *et al*. 2019).

Although the roles of Zn as a component of enzymatic and structural proteins and its involvement in several physiological processes are well documented (Coleman 1998; Duffy 2007; Cabot *et al*. 2019), mechanisms through which it operates in plant defence are not yet well known. Thus, with the aim of elucidating some of these mechanisms and their role against fungal pathogens with different lifestyles, we assessed the impact of the Zn nutritional imbalance, both deficiency and excess, on some physiological parameters and the severity of infections with the necrotroph *B. cinerea* or the biotroph *G*. *cichoracearum* on *A. thaliana* Col‐0.

At 7 dpt, the leaf Zn concentration (49 mg kg^−1^ DW) in plant supplied with Zn‐sufficient solution was comparable with that reported in literature for *A. thaliana* shoots (51 mg kg^−1^ DW) (Peer *et al*. 2003). Moreover, according to Helfenstein *et al*. (2015), there were significant differences among treatments, with leaf Zn concentration reflecting the dose in the nutrient solution. Co‐administration of SA significantly affected leaf Zn content in plants with Zn‐sufficient or Zn‐excess solutions, with a slight increase in plants that had received the physiological dose and a considerable reduction in the other Zn treatments. Indeed, SA, in addition to being a hormone involved in plant defence responses, also affects nutrient element uptake (Lu *et al*. 2018; Mabrouk *et al*. 2019; Sharma *et al*. 2020). Some authors found that exogenous application of SA reduced Zn content in leaves (Wang *et al*. 2011), whereas other studies found no significant change in the mineral concentration (Shi & Zhu 2008), indicating that the precise role of SA, as well as its mechanism of action on the regulation of nutrient uptake, remain unclear. These differences in leaf Zn concentration in plants supplied with the different Zn doses, with or not SA, are related to differences in some parameters subsequently investigated.

The first parameter was pigment content, the second was lipid peroxidation. These analyses were carried out to highlight plant response to oxidative stress generally induced by nutrient imbalances, both Zn deficiency and excess. The carotenoid content was evaluated for potential role as an efficient antioxidant that protects the photosynthetic apparatus (Strzałka *et al*. 2003). A reduction in carotenoids with Zn deficiency or excess, and simultaneous reduction in chlorophyll, enhances the accumulation of reactive oxygen species (ROS), as reported in plants such as mandarin orange, red cabbage and tea (Subba *et al*. 2014).

The reduced chlorophyll content observed in *Arabidopsis* under both Zn‐deficient and Zn‐excess is similar to that reported in the literature. Indeed, Zn is involved in the development and functioning of chloroplasts (Broadley *et al*. 2007; Hansch & Mendel 2009; Sharma *et al*. 2013) and reduced chlorophyll synthesis, altered chloroplast structure and inhibition of photosynthesis are known effects of Zn deficiency (Chen *et al*. 2008; Fei *et al*. 2016; Roosta *et al*. 2018). Also, high Zn concentration can be phytotoxic, causing chlorosis and degradation of chloroplasts (Chaney 1993). On the other hand, Zn affects the uptake and transport of other micronutrients, such as magnesium and iron, which are directly involved in chlorophyll production (Chaney 1993). Thus, plants treated with excess Zn show iron deficiency (Haydon *et al*. 2012; Shanmugam *et al*. 2012; Briat *et al*. 2015). Wang *et al*. (2016) found, in *A. thaliana* Col‐0, that carotenoid content was not affected by Zn treatments at levels similar to those used in our experiments. Thus, the protective role of Zn as an antioxidant in photosynthesis varies with plant species and Zn dose.

Addition of SA significantly affected both chlorophyll and carotenoid concentration, increasing them only in plants given Zn excess. Similar results were reported in *Targetes erecta* L., where the positive effect of SA co‐supply on the level of chlorophyll and carotenoids increased proportionally with the increasing dose of the micronutrient (Choudhary *et al*. 2016). This positive effect could be related to the SA‐mediated activation of antioxidant enzymes and biosynthesis of secondary metabolites with stress‐protective roles (Kim *et al*. 2009; Al‐Whaibi *et al*. 2012; Sadeghi *et al*. 2013).

For lipid peroxidation, as a marker of oxidative stress, we measured MDA rather than the concentration of H_2_O_2_ or the superoxide ion, which are transient and therefore poorly suited to assess oxidative damage after 7 days of Zn deficiency or excess. The increased content of MDA in Zn‐deficient solution indicated oxidative stress. Production of ROS under Zn‐deficiency is well‐known, and due to iron‐mediated free radical formation (Cakmak 2000). Indeed, leaf iron accumulation promotes ROS formation, especially the hydroxyl radical (^.^OH), leading to oxidative damage to cellular components in Zn‐deficient plants (Cakmak 2000; Andresen *et al*. 2018). Plants can reduce ROS damage by activating detoxifying enzymes, such as superoxide dismutase (SOD; EC 1.15.1.1), ascorbate peroxidase (APX; EC 1.11.1.11), catalase (CAT; EC 1.11.1.6), peroxidase (POX; EC1.11.1.7) and glutathione reductase (GR; EC 1.6.4.2) (Bowler *et al*. 1992; Celik & Atak 2012). Mitigation of oxidative stress is closely related to the role of SOD, a group of rapid enzymes present in most cell compartments (Alsher *et al*. 2002). Reduced activity of Cu/Zn‐SOD, a SOD that binds both copper and Zn as cofactors, has been reported in several species under Zn deficient conditions (Sharma *et al*. 2004; Tewari *et al*. 2008, 2019; Saibi & Brini 2018), and results in increased MDA and H_2_O_2_ content (Sharma *et al*. 2004). Differences in plant antioxidant capacity and oxidative stress induction have also been reported under Zn‐excess conditions (Jain *et al*. 2010; Feigl *et al*. 2015). However, in our study, 25 µM Zn did not affect the MDA content. Effectively, the Zn concentration used in our experiments, although several times higher than the physiological level, was lower than levels reported in the literature (50–800 µM) as able to mimic Zn excess (Jain *et al*. 2010; Feigl *et al*. 2015). Thus, the Zn concentration here used may be too low to determine significant accumulation of ROS, but sufficient to induce antioxidant systems that provide an effective counter to oxidative stress. However, the purpose of our work was to assess the impact of different concentrations of Zn in defence against fungal pathogens, without inducing excessive toxicity.

In plants supplied with Zn‐deficient solution, addition of SA significantly reduced oxidative stress by lowering MDA to the control value, thus demonstrating the protective role of SA against ROS accumulation, as previously reported for photosynthetic pigments.

Here, the different Zn concentrations also affected responses to both necrotrophic and biotrophic fungal pathogens. The higher susceptibility of *A. thaliana* both under Zn deficiency and Zn excess to the necrotrophic pathogen *B. cinerea*, with Zn deficiency having a larger impact, could be related to the induction of oxidative stress in Zn‐deficient plants, where the increase in lipid peroxidation and subsequent cellular damage benefits the necrotrophic fungus. Indeed, it has been reported that *B. cinerea* bypasses the oxidative burst of the host plant and exerts virulence directly proportional to the intensity of the host defence response (Tudzynski & Kokkelink 2009). Moreover, Govrin & Levine (2000) found that, during its infection process, *B. cinerea* directly causes cell death and induces a hypersensitive response (HR) in *Arabidopsis* and that *Arabidopsis* HR‐deficient mutants are resistant to *B. cinerea*.

Resistance to necrotrophs, including *B. cinerea*, is usually associated with activation of the JA‐resistance pathway, as highlighted by the increased transcription of JA‐responsive genes, such as plant defensins (Penninckx *et al*. 1998; Sham *et al*. 2019). In the hyperaccumulator plant *Thlaspi caerulescens* J. & C. Presl and *Arabidopsis halleri* O’Kane and Al‐Shehbaz, Zn deficiency and/or Zn excess strongly induce the expression of the *PDF* gene family (Mirouze *et al*. 2006; van de Mortel *et al*. 2006). However, in *A. thaliana* both Zn deficiency and Zn excess reduced expression of *PDF* genes (van de Mortel *et al*. 2006). Similarly, in the present work, a weak, although not significant, decrease in *PDF 1.2* transcripts was observed both in Zn deficient and Zn excess plants, in comparison with control, which was supplied with a physiological Zn dose. The lack of *PDF 1.2* induction could at least partially explain increased susceptibility to the fungus observed here in plants that received an unbalanced Zn supply. Co‐supply with SA greatly increased transcript accumulation of this gene in all treatments, increasing resistance to the fungus only in Zn‐deficient plants. The observed induction of *PDF 1.2* after SA treatment may seem surprising, since *PDF1.2* induction is mainly linked to the JA/ET resistance pathways (Pangesti *et al*. 2016) that are antagonized by the SA resistance pathway. However, synergism has been reported for SA and JA/ET defence pathways (Beckers & Spoel 2006; Mur & Kenton 2006), and recent studies demonstrated that SA produced during early stages of the effect‐triggered immunity (ETI) is a necessary signal not for suppression but rather for stimulation of JA signalling (Liu *et al*. 2016). Probably, the role of SA as a signal triggering the antioxidant systems is particularly effective where the level of oxidation is high, as in Zn‐deficient plants.

With regard to *G*. *cichoracearum*, increased susceptibility to the fungus was observed with increasing Zn dose. The lower susceptibility of plants supplied with Zn ‐deficient solution could be due to the higher oxidative stress, making the cellular environment unsuitable for this fungus with a biotrophic lifestyle. Indeed, it is well known that ROS accumulation typical of the HR response is specifically associated with plant resistance to biotrophic pathogens (Balint‐Kurti 2019). The higher susceptibility of plants supplied with Zn excess, might indicate that the used concentration (25 µM) did not limit the development of *G. cichoracearum*. Moreover, the more severe infection could be related to the lack of oxidative burst caused by activation of antioxidant systems in the presence of Zn concentrations higher than the physiological concentration. This response to Zn excess may have weakened the defences induced by the pathogen, such as ROS accumulation and related activation of defence genes. Indeed, it has been reported that, in comparison with the non‐hyperaccumulator *A. thaliana*, in hyperaccumulator *Noccaea caerulescens* (C. Presl) a high Zn concentration resulted in reorganization of the defence pathway against *Pseudomonas syringae* pv. *maculicola*, with the loss of the oxidative burst and downstream responses, such as induction of SA‐responsive pathogenesis‐related (*PR*) genes (Fones *et al*. 2013). This supports our results, where SA addition to the nutrient solution significantly reduced infection by *G. cichoracearum* at all applied Zn doses.

The critical role of SA as a hormonal signal for defence against biotrophic pathogens, such as *Golovinomyces* spp. (Ding & Ding 2020), was also confirmed by its effect on callose formation, which is induced in response to fungal infection (Jacobs *et al*. 2003). We have shown that different doses of Zn did not influence the accumulation of callose, which was instead strongly stimulated by SA supply.

## CONCLUSION

This work confirms the key role of Zn in plant metabolism and defence, and demonstrates that deviation from the physiological concentration, both deficiency and excess, can significantly affect plant physiology and response to fungal infections, varying in relation to the pathogen lifestyle. Hence, Zn fertilization of crops in order to supplement this micronutrient in the human diet should be carefully evaluated because it can determine the success or failure of the crop, as well as the function of stress factors with which it might interact. From a practical point of view, the effect of mineral nutrition on plant–pathogen interactions should be investigated species‐by‐species and pathogen‐by‐pathogen so as to use the appropriate mineral nutrition as a plant protection measure.

## AUTHOR CONTRIBUTIONS

MQ and LE contributed to conceptualization, methodology, analysis, investigation, data curation, writing and funding acquisition. ET and RDA contributed to methodology and analysis of data related to zinc level in leaves.

## CONFLICT OF INTEREST

All authors confirm that they have no conflict of interest to declare.
